# Transcriptional Control: A Directional Sign at the Crossroads of Adult Hepatic Progenitor Cells' Fates

**DOI:** 10.7150/ijbs.93739

**Published:** 2024-06-24

**Authors:** Chenhao Xu, Xixi Fang, Yisu Song, Ze Xiang, Xiao Xu, Xuyong Wei

**Affiliations:** 1 Zhejiang University School of Medicine, Hangzhou First People's Hospital, Hangzhou 310006, China.; 2 Zhejiang University School of Medicine, Hangzhou 310058, China.; 3 Key Laboratory of Integrated Oncology and Intelligent Medicine of Zhejiang Province, Hangzhou 310006, China.; 4 Hangzhou Normal University, Hangzhou 310006, China.

**Keywords:** Hepatic progenitor cells, Transcription factors, Liver injury, Liver regeneration, Fate regulation.

## Abstract

Hepatic progenitor cells (HPCs) have a bidirectional potential to differentiate into hepatocytes and bile duct epithelial cells and constitute a second barrier to liver regeneration in the adult liver. They are usually located in the Hering duct in the portal vein region where various cells, extracellular matrix, cytokines, and communication signals together constitute the niche of HPCs in homeostasis to maintain cellular plasticity. In various types of liver injury, different cellular signaling streams crosstalk with each other and point to the inducible transcription factor set, including FoxA1/2/3, YB-1, Foxl1, Sox9, HNF4α, HNF1α, and HNF1β. These transcription factors exert different functions by binding to specific target genes, and their products often interact with each other, with diverse cascades of regulation in different molecular events that are essential for homeostatic regulation, self-renewal, proliferation, and selective differentiation of HPCs. Furthermore, the tumor predisposition of adult HPCs is found to be significantly increased under transcriptional factor dysregulation in transcriptional analysis, and the altered initial commitment of the differentiation pathway of HPCs may be one of the sources of intrahepatic tumors. Related transcription factors such as HNF4α and HNF1 are expected to be future targets for tumor treatment.

## Introduction

The liver is an amazing internal organ with multiple complex functions and therefore deserves extensive exploration. It performs partial hematopoietic and immunological functions, participates in metabolism and medication biotransformation, and is a key location for manufacturing the body's albumin and coagulation components. Unlike other internal organs, the liver has an impressive capacity to regenerate and maintain its vital functions after enduring various kinds of injuries. Liver regeneration is a well-orchestrated process that includes numerous cellular modifications and remodeling brought on by extracellular stimuli. Under normal conditions, the majority of hepatocytes are in the functional state (G0 phase) 1, 2. The surviving hepatocytes quickly enter a condition of replication after liver injury or partial hepatectomy. Hepatocyte proliferation mediates liver regeneration, which is quick and efficient and is regarded as the first line of defense against liver injury. Hepatocyte regeneration mediated by hepatic progenitor cells (HPCs), also referred to as the secondary line of defense for liver regeneration, becomes the main body of liver compensation when a significant proportion of hepatocytes die or replication is impeded in severe liver illness. The significance of HPCs in clinical settings has never stopped being investigated. More speedy regeneration might take place if early progenitor cells in the injured liver can be found and encouraged to multiply. This would increase our knowledge of liver physiology and make it easier to potentially develop novel treatments for liver diseases.

HPCs express markers of both bile duct epithelial cells and hepatocyte lineages with bidirectional differentiation potential. In the course of HPCs differentiation, intricate signaling networks and intercellular interactions affect the transcriptional profile of the cells. Signaling molecules can regulate transcription factor activity directly or indirectly to control HPCs fate selection. Transcription factors bind to the regulatory regions of their target genes at specific times to activate transcription, resulting in corresponding gene expression patterns for differentiation of the target cells. In this review, we summarized the functions of the major transcription factors involved in the processes of proliferation, differentiation, and self-renewal of HPCs based on the context of HPCs' plasticity. The review brings some paradigms and reflections on the study of cell fate transduction, which we hope will further deepen the exploration of the precise regulation of the fate of HPCs.

## HPCs and their niche

In 1956, an oval cell with a large nucleoplasmic ratio was first identified by researchers in a rat model of liver cancer 3, which served as the prototype for the recognition of HPCs. Since then, the term “oval cell” has been used in the literature as a synonym for HPCs. However, for a long time, the properties of such cells were not extensively studied and their stemness or even their existence was questioned, probably due to the imperfections of certain animal models. Partial hepatectomy (PHx) is considered an excellent model for liver regeneration, but since it does not cause damage to residual liver tissue, therefore cannot reflect the body's true pathological changes, such as certain inflammation and fibrosis, which makes it difficult to detect the “oval cells”. In fact, these cells have been demonstrated to exist in animal studies 4, 5, and are believed to possess the following qualities: (1) high growth potential cloning; (2) the capacity to induce differentiation into hepatocyte and biliary cell lineages; and (3) the capacity to repopulate the liver following transplantation 6. To standardize the designation of the stem cell population activated by various liver diseases, “Hepatic Progenitor Cells” is an appropriate descriptive term.

Tracking at the single-cell level is required to determine the biological characteristics of HPCs at the macroscopic level. Stem cell markers such as CD133, EpCAM, CD24, CD44, Trop2, A6, Mic1-1c3, Sca-1, Lgr5, biliary cell markers such as Sox9, Foxl1, Opn, CK19, and hepatocyte markers such as HNF4α, CK8 have all been identified 6-8. Most HPCs markers lack specificity and are easily confused with other cells because they may already be expressed on other cells for a single marker, or certain molecular markers may be up/down-regulated by obtaining a specific gene expression profile in certain inflammatory environments 9. This variability is difficult to predict. Therefore, we prefer to use dual markers of bile duct epithelial cells and hepatocytes or stemness markers as characteristics for HPCs, with the overlay of multiple molecular markers being more convincing. Biomarkers commonly used by researchers for presumptive HPCs are displayed in **Table 1**.

**Figure 1:**
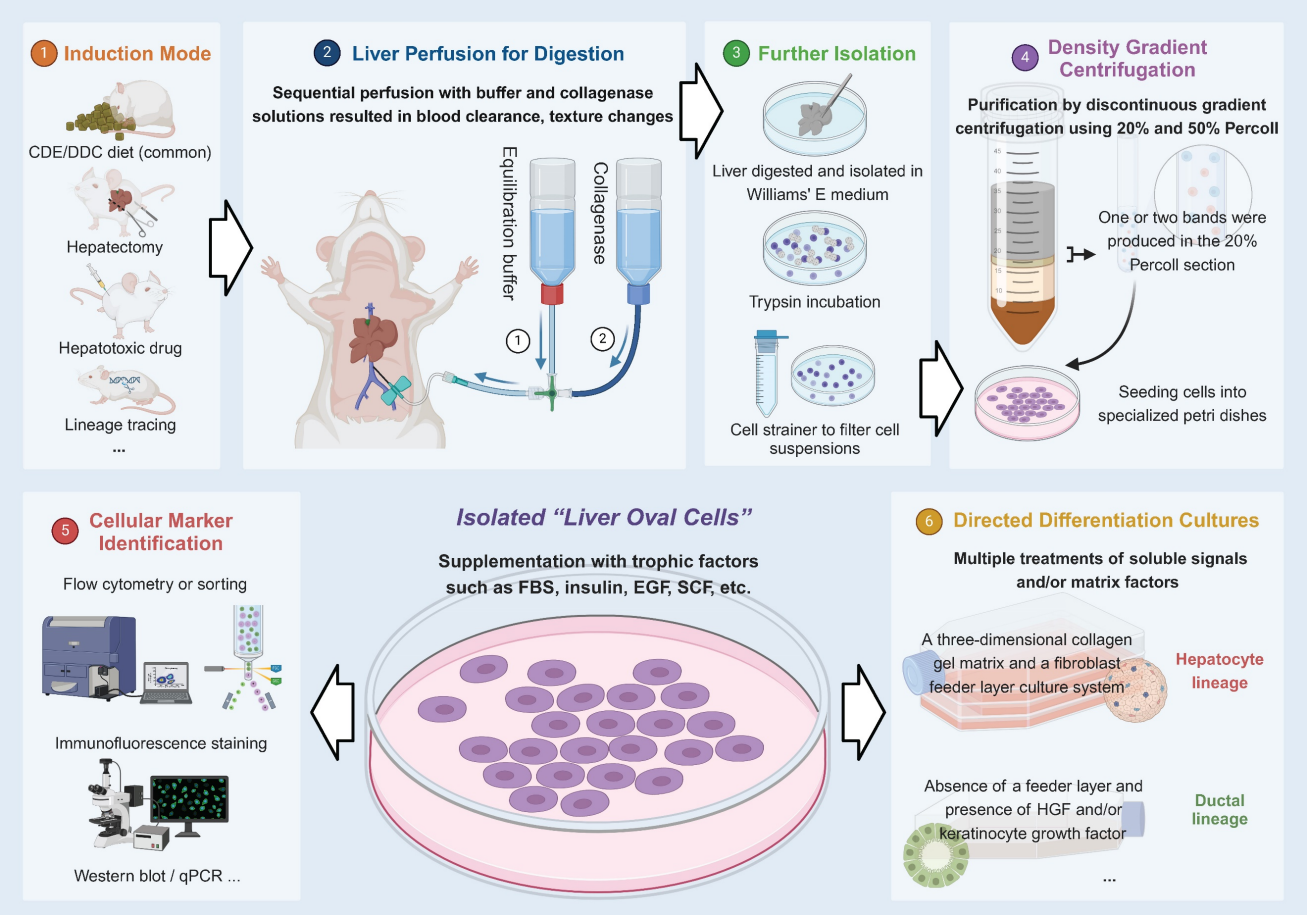
Isolation process of “liver oval cells”.

For the in-depth study of HPCs, isolation and *in vitro* culture is technique. In 2007, a team successfully isolated this type of bipotential stemness population (liver oval cell) from adult mice fed with a choline-deficient, ethionine-supplemented (CDE) diet, and then cultured *in vitro* 20. In subsequent studies, the cells preserved progenitor cell characteristics in long-term *in vitro* cultures, and there was no spontaneous malignant transformation after prolonged culturing, so this could be a source of expandable stem cells in the future 20-23. **Figure 1** depicts this process.

**Figure 2:**
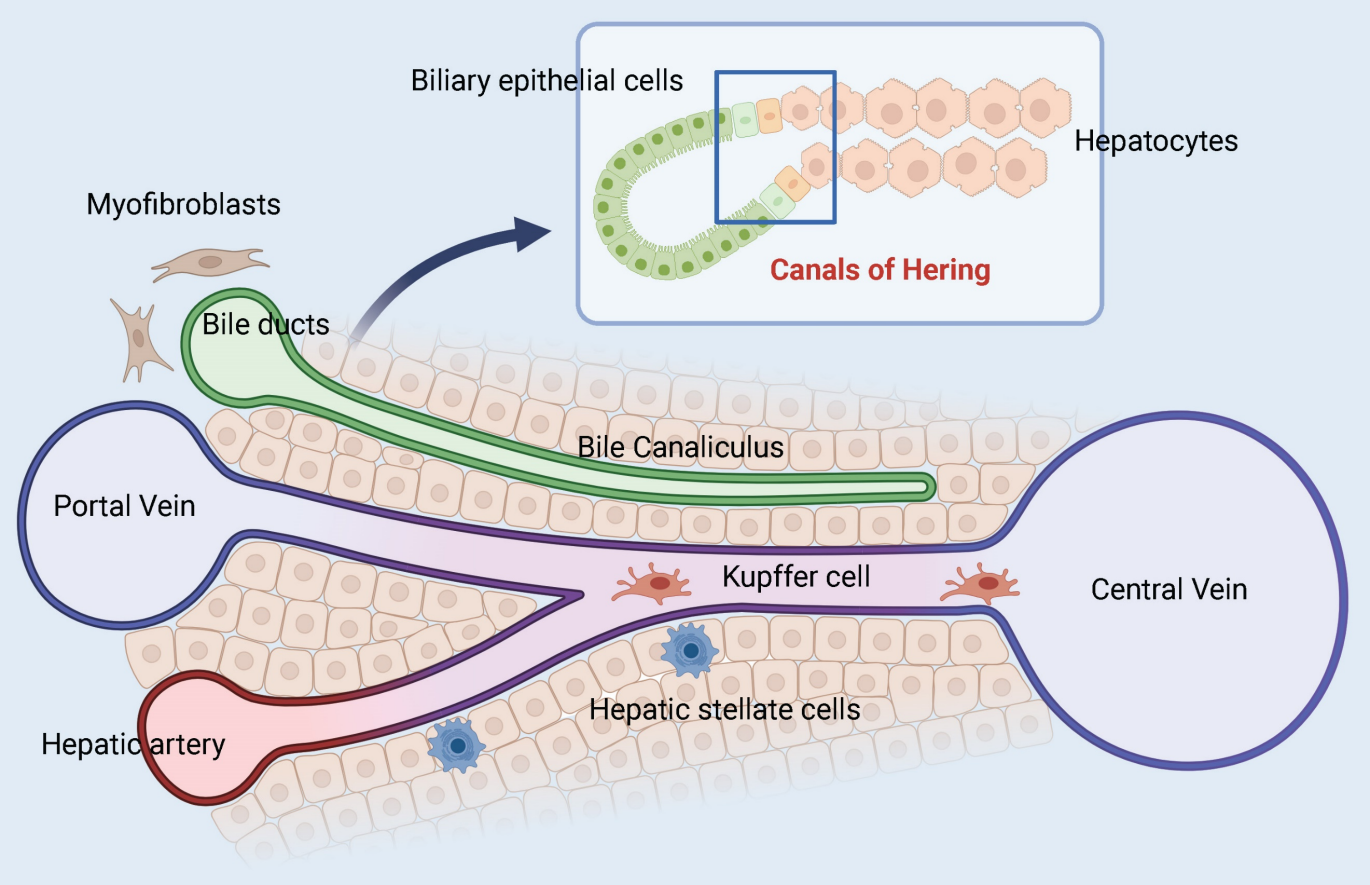
** Model of the adult hepatic progenitor cells niche.** The interface between the end of the bile duct and the origin of the hepatic plate is called the canals of Hering. Different cell types, ECM components, and signaling streams converge here to help maintain the plasticity of adult HPCs.

In 1978 Raymond Schofield pioneered the concept of stem cell niches, which are specific regulatory microenvironments 24. The niches are made up of various cells, extracellular matrix, cytokines, and communication signals that work together to keep the balance between differentiation and self-renewal of stem cells. HPCs formed by stimulation of hepatic injury are found at the canals of Hering (CoH), which connect the hepatocytes and the biliary tree **(Figure 2)** 25, 26. This distinct anatomical location corresponds to the physiological function of the HPCs, which upon signal induction differentiates into hepatocytes or bile duct cells and serves as the physiological link between the two. In addition, the space of Disse, a unique perisinusoidal space in the liver parenchyma, may also provide a temporary niche for stem cells 27. Other reports indicate that endothelial cells in the central vein can provide Wnt signals to surrounding cells, which constitutes their niche. A population of Axin2(+) cells that can self-renew or differentiate into other hepatocytes in the hepatic lobules was found nearby, which were considered hepatic stem/progenitor cells 28. However, recent research has shown that all hepatocytes can upregulate Axin2 and Lgr5 after injury and denies the signaling dominance of the pericentral venous region and the stemness of the Axin2(+) cell population 29. Indeed, the establishment of the HPCs niche is heavily reliant on identifying the signaling pathways involved, such as Wnt/β-catenin 30, 31, Notch 32, 33, Hedgehog 34, Hippo/YAP 35, HGF/c-Met 36, TWEAK 37, and others. These signals, to varying degrees, regulate the survival, proliferation, migration, and differentiation of HPCs and are critical for the niche's function.

## Activation and Differentiation of Adult HPCs

Histologically, the portal vein, hepatic artery, and bile duct form the portal triad, and are separated from the central vein by a plate-like structure composed of hepatocytes. Theoretically, in response to acute or chronic liver injury, cells with bile duct markers infiltrate from the portal vein region into the injured area of liver parenchymal cells, with concomitant expansion of the underlying stem cell niche in the portal region, a phenomenon known as the ductular reaction (DR) 38, which is pathologically described as biliary hyperplasia with inflammatory cell infiltration in the portal vein region 39. Inflammatory conditions in the DR drive the transformation of a subpopulation of biliary epithelial cells (BECs) with potential stemness to HPCs (the currently recognized sources of HPCs) and thus play a role in injury repair 19. This is a complex and multi-stage process that involves changes in the markers of progenitor cells. For example, when hepatocyte-mediated regeneration is impaired, the expression of CK19 is progressively attenuated 19. In addition, under the influence of defined signaling streams such as Hippo/YAP, hepatocytes can also de-differentiate into stem-like cell populations, which have been named liver-progenitor-like cells (LPLCs)^16^, ^17^. Its origin may take many forms or be defined and named differently in different studies. As mentioned previously, HPCs is an appropriate name to unify the population of stem cells activated in various liver diseases, which led us to think of HPCs as a population of stem cells with bidirectional differentiation potential, whose multiple fate outcomes and high plasticity are of interest for our study.

Study has shown that the activation of HPCs has a positive correlation with the degree of liver injury and the level of DR response, which is the proliferative form of HPCs. In humans, extensive HPCs activation requires a 50% threshold for hepatocyte loss 40. Zhou *et al.* revealed by NCAM, CK19, and HepPar1 staining that DR embedded in cirrhotic tissues had a distinct hepatocyte and biliary tract bipolar structure 41. Charles *et al.* used a multi-marker approach to detect FoxA2, HepPar1, albumin, CK19, and miRNA expression to track differentiation in the stem cell lineage during end-stage cirrhosis, indicating a bifurcation direction of HPCs differentiation 42.

In response to a changing environment, the plasticity of HPCs differentiation remains in a dynamic balance between the initial commitment of these cells and their environmental adaptability. This relies in part on the combination of inter-crosstalk cell signaling networks with a group of inducible transcription factors, including FoxA1/2/3, YB-1, Foxl1, Sox9, HNF4α, HNF1α and HNF1β, to enable the appropriate genetic information to be transcribed, facilitating the transition of HPCs in both directions. Other somatic cells (like fibroblasts), when forced to express a combination of these factors, can also develop into “bipotential hepatic stem cells” 43, 44.

## FoxA1/2/3 and Inhibitory Cell Specification

The forkhead box protein A (FoxA) transcription factor family consists of three family members FoxA1, FoxA2, and FoxA3 (or HNF3α, HNF3β, and HNF3γ) 45. These proteins contain a helix-loop-helix DNA-binding structural domain flanked by two polypeptide chains and two conserved trans-activating structural domains that are essential for FoxA to localize binding to target gene nuclei 46. The FoxA family is involved in the transcriptional regulation of many important genes expressed in embryonic development and organogenesis in the liver, pancreas, intestine, lung, thyroid, and prostate 47-51, and also plays an important role in the regulation of HPCs fate.

FoxA2 has the most significant effect in the family, and it is downregulated in liver tissue from mice with cholestatic liver injury caused by carbon tetrachloride (CCl_4_) injury or bile duct ligation (BDL) 52, 53. In contrast, overexpression of FoxA2 in hepatocytes inhibited hepatocyte apoptosis in mouse fibrotic livers 52. FoxA2 can be used as a marker for the stem cell profile of HPCs 42. Studies have shown that FoxA2 has an inhibitory effect on the proliferation of HPCs during chronic liver injury from different causes. The molecular mechanism of the effect of FoxA2 on HPCs depends on the reduction of hexokinase 2 (HK2) gene transcription, protein expression, and enzyme activity. In addition, FoxA2 downregulates the expression of growth-promoting genes in the phosphatidylinositol 3-kinase (PI3K)/protein kinase B (Akt) signaling pathway, such as Lama1, IL-6, and PI3K, which reduces Akt phosphorylation and HK2 activity, thereby decreasing aerobic glycolysis and proliferation of HPCs (from rats on CDE diet) 54. Recent studies have also highlighted the potent intervention of FoxA1/2 in cellular metabolism. Knocking down of FoxA1/2 leads to global reprogramming of the cellular differentiation state and metabolism, hindering the differentiation of human stem cell-derived HPCs 55. It is the inhibition of proliferation and metabolism by FoxA1/2 that exemplifies its critical role in maintaining self-renewal during the resting state. FoxA1/2 may be the cornerstone of HPCs' existence, and like most inhibitory factors, it maintains the transition state of HPCs by forming a cellular specification, which is why FoxA is indispensable in numerous studies of reprogramming somatic cells into “HPCs” 43, 44. Although the role of FoxA1/2 in HPCs has not been fully elucidated, it has suggestive implications for HPCs differentiation, metabolism, regeneration, and disease states such as cancer and liver fibrosis.

We want to emphasize that individual transcription factors are not independent, but rather part of a transcriptional regulatory network that exerts influence. Depletion of Y-box binding protein-1 (YB-1) in HPCs in chronic liver fibrosis models significantly inhibits its epithelial-mesenchymal transition (EMT) and suppresses Akt phosphorylation *in vitro* and *in vivo* 56. FoxA2 may be involved in this process by downregulating the Akt signaling pathway to disrupt YB-1 nuclear translocation and achieve the phenotype. In addition, FoxA2 was associated with the initiation expression of FoxA1, HNF1β, HNF4α, and other important transcription factors of HPCs 55. FoxA3 plays a role in compensating for the loss of FoxA1/2. The FoxA triple null mouse model suggests that FoxA protein maintains the adult hepatic regulatory network by facilitating the binding of HNF4α to enhancers 57.

## YB-1 and Autophagy Flow

The Y-box binding protein (YB protein) family consists of three members, YB-1, YB-2, and YB-3, which are highly conserved in terms of cold shock domain (CSD) but have different C-terminal domain (CTD) sequences as specific markers and belong to a family of evolutionarily conserved CSD proteins 58. Among them, Y-box protein-1 (YB-1), which regulates DNA and RNA binding activity and functions as a nucleic acid chaperone, is a well-known translational and transcriptional regulator. YB-1 is involved in cell cycle progression, DNA and RNA repair splicing, cell proliferation, and invasion by translocating to the nucleus and transcriptionally regulating target genes 59.

It has been shown that YB-1 ablation dramatically prevented the amplification of HPCs and suppressed fibrosis in a mouse model of selective YB-1 knockout that was fed a DDC or a CDE diet 60. Matrix metalloproteinases (MMPs) are responsible for degrading the extracellular matrix (ECM) by catabolizing various proteins in cellular structures, and overexpression of various MMPs leads to ECM catabolism and promotes EMT 61. YB-1 binds directly to the Mmp9 gene promoter and positively regulates the EMT of HPCs. The ductal response in chronic liver injury releases TGF-β to induce AKT phosphorylation and YB-1 nuclear translocation in HPCs, activating the expression of proliferation-related and fibrosis-related genes (Acta2, Col1a1, Col3a1), thus promoting the expansion of HPCs and exacerbating liver fibrosis. However, the YB-1 ablation model inhibits the dual pathway of proliferation and fibrosis to alleviate this pathological process 56. Kupffer cells and inflammatory cells may be linked to the degree of TGF-β release after chronic liver damage. The identification of the TGF-β/YB-1/Atg7 axis reveals another way in which HPCs worsen liver fibrosis. TGF-β induces YB-1 nuclear translocation, targets binding to autophagy-related gene 7 (Atg7), and upregulates its transcriptional level to initiate autophagic flow in HPCs, which is essential for proliferation and differentiation 18. High levels of autophagic activities, which support self-renewal and differentiation into hepatocytes via the Wnt/β-catenin signaling pathway, prevent drug-induced cellular senescence and may lessen HPCs damage during liver injury to aid in liver regeneration, are necessary for HPCs 62, 63.

## Foxl1 and HPCs Proliferation-Associated Signaling

Foxl1, another member of the DNA-binding transcription factor family of forkheads, is located in the Fox gene cluster on chromosome 16q24.1 and has a winged-helix DNA-binding domain 64. It is expressed in numerous human organs and is known to be an important source of niche signaling in certain stem cells, such as the intestine 65, 66. In oncology studies, there is a positive crosstalk between Foxl1 and downstream Wnt/β-catenin signaling activation 67. Although the detailed molecular mechanism is not very clear in HPCs, based on the significance of the Wnt/β-catenin pathway for HPCs 30, 62, 68, 69, Foxl1 may assume part of the effect in the pathway. Foxl1 has been recognized as a specific lineage marker for bipotential hepatic progenitor cells in the adult liver and is commonly used for genetic lineage tracing to study the activation and differentiation status of hepatic progenitor cells during liver injury 14, 70. Hepatic cells with progenitor-like features (iHepL cells) generated by transcription factor set induction do not express pluripotency markers but have high levels of Foxl1 and Cd24a, ductal cell markers, and immature hepatocyte markers, and therefore Foxl1 is closely related to the intrahepatic stem cell population 71.

Foxl1 is closely related to the hedgehog signaling pathway 65, 72. In a DDC model study, Foxl1 expression was enriched around the portal triad and was absent in parenchymal cells. HPCs expressing Foxl1-Cre are in close association with periportal fibroblasts and hepatic stellate cells and have a paracrine role 14. Foxl1 may be involved in maintaining HPCs viability and proliferation by forming crosstalk connections with fibroblasts with the help of hedgehog signaling, and such intercellular communication may also be one of the reasons for the exacerbation of liver fibrosis caused by HPCs proliferation. In addition, Shin *et al.* designed a refined transgenic mouse model that can ablate Foxl1-expressing HPCs and their progeny by labeling yellow fluorescent protein (YFP) tracking and administering diphtheria toxin (DT). Following ablation, mice on the CED diet showed a dramatic increase in lipid accumulation and a markedly impaired recovery of liver function. Foxl1+ HPCs or their progeny may be required for BECs and hepatocyte development after CDE diet-induced injury 73.

## Sox9, Hippo/YAP, and Notch Signaling

Sex-determining region Y-related high mobility group box9 (Sox9) is a class of transcription factors essential for embryonic development. It is expressed in several tissues and organs during embryogenesis, including chondrocytes 74, heart 75, and pancreas 76. In the liver, it mainly regulates the development of the biliary system and determines the timing of intrahepatic bile duct formation 77. During embryonic development, Sox9 maintains cells in an undifferentiated state. Under normal conditions, Sox9 is mainly expressed in BECs and generally remains silent in other cells. When the liver injury occurs, Sox9 may act on hepatocyte extracellular matrix genes, leading to extracellular matrix deposition 78, 79 and induction of non-coding RNA H19 by binding to the conserved promoter region of the H19 gene, while participating in hepatocyte apoptosis and liver fibrosis 80.

The Hippo/YAP signaling pathway is one of the pathways that activate the initiating effects of Sox9. Yes-associated protein 1 (YAP) and transcriptional co-activators with PDZ-binding motifs (TAZ) maintain organ growth and cell plasticity by regulating the expression of proliferation, differentiation, and metabolism-related genes and are downstream effectors of the Hippo pathway 81. Yimlamai *et al.* showed that on a Dox-induced YAP tracer model, the elevated YAP activity clarifies the identity of HPCs, and its ectopic activation in hepatocytes leads to their dedifferentiation and acquisition of progenitor cell characteristics. The differences in transcript levels of YAP present between hepatocytes and progenitor cells can determine the fate of different cells. Gene set enrichment analysis (GSEA) showed that the genetic markers of HPCs are similar to those of YAP transgenic cells and that YAP activation would lead to their proliferative response 35. Notably, Sox9 is a downstream target of YAP protein activation and contributes to the progression of the ductal response 82, which may be the initiating source of YAP-promoted HPCs proliferation. Huh-7 cell models co-expressing Sox9 and YAP show a negative feedback pathway between the two and that Sox9 may inhibit YAP transcription and thus interfere with HPCs. In addition, YAP-Sox9 signaling is indispensable in the reprogramming transition from mature hepatocytes to hepatic progenitor cells, hepatocarcinogenesis, and the formation of tumor heterogeneity 83. Lijian Hui's team found that LPLC formation was reduced in Yap-KO mice on a DDC diet, and that Yap plays a critical role in triggering LPLC formation under liver injury 16.

Sox9 is a direct target of the Notch signaling pathway, and Notch1 can trigger its expression by binding directly to the Rbpj binding site in the Sox9 promoter 84, while the Notch signaling pathway is also an important functional effector downstream of Hippo/YAP 35, implying that there is multiple signaling crosstalk for the regulation of HPCs. The directional differentiation of HPCs by Notch is currently the most clearly studied among the many interleaved signals, and the direct effect of signal flow is the most significant. For example, the expression of Notch ligands by myofibroblasts promotes BECs selection of HPCs, or macrophages derive classical Wnt signaling after phagocytosis of hepatocytes fragments that impairs Notch to create the specification of hepatocytes (from CDE/DDC diet mice) 33; also, in inflammatory injury with high levels of TNFα, macrophages become determinants that support the expansion of HPCs 85. Similar results were found in a zebrafish model with liver fibrosis, where antagonism of Notch transduction was expressed through the inhibition of Sox9, which blocked BECs proliferation and promoted HPCs to hepatocyte differentiation *in vitro* 86. Administration of DAPT (Notch signaling inhibitor) in a BDL rat model resulted in a significant reduction in the expression of OV6, Sox9, and EpCAM, reducing the differentiation of HPCs into bile duct cells to prevent cholestatic liver fibrosis 87. The study of Ko *et al.* in 2019 demonstrated a very interesting phenomenon where a decrease in the activity of the same factor resulted in a splitting mechanism and they showed a more detailed mechanism of action of the Notch-Sox9 axis 88. Following transgenic disruption of histone deacetylase Hdac1 activity in zebrafish, Sox9b mRNA transcript levels were upregulated to inhibit HPCs differentiation into hepatocytes selection, or Cdk8 mRNA transcript levels were increased (responsible for negatively regulating Notch signaling) to reduce HPCs differentiation toward BECs. These two opposing transcriptional regimes ultimately act on three key genes of Notch signaling (CDK8, FBXW7, and Notch3, with studies showing that low expression of Fbxw7 predisposes HPCs to biliary lineage differentiation) to achieve a targeted fate shift. Thus, we postulate that the Notch-Sox9 signaling axis is a fate regulator of HPCs differentiation toward the biliary tract spectrum, and the expression of Sox9 in hepatocytes is considered to be in a bipotent state with progenitor-like characteristics.

## HNF4α regulates the differentiation of HPCs to hepatocytes

Hepatocyte nuclear factor 4 α (HNF4α) is often used as a candidate marker for HPCs. HNF4α belongs to the nuclear hormone receptor transcription factor family and has been shown to directly regulate the expression of a large number of developmental genes in the liver 89, 90. Genome-wide promoter studies in adult liver have shown that HNF4α binds more than 40% of active gene promoters 91 and occupies active enhancers in hepatocytes to increase transcription of intracellular genes 92. HNF4α is encoded by two developmental regulatory promoters (P1 and P2). Two transactivating functional domains (AF1 and AF2) are located at the N-terminal and C-terminal ends of the protein, respectively, and a ligand binding domain (LBD) is present in its vicinity for additional regulation of protein activity. In addition, HNF4α has a specific inhibitory structural domain F, which is not found in other HNF family members 93. As early as 1994, Nagy found in an experimental model of oval cell proliferation and differentiation that HNF4 was not expressed in the early stages of oval cell proliferation. Liver-enriched transcriptional factors such as HNF1 α, HNF3 α, HNF4, and C/EBP became highly expressed in the later stages, with HNF4 being the first to be expressed when oval cells differentiated into hepatocytes 94. This expression, however, was later found to be dependent on the involvement of the forkhead box protein H1-Sma and Mad homolog 2/3/4 transcription factor complex, which is promoted by TGF-β superfamily member activin 95. Currently, the effect of HNF4 on the differentiation of HPCs into hepatocytes is gradually being confirmed. In a study by Razvan *et al.*, Epithelial morphology, secretory function, and metabolic activity of hepatocytes were repeatedly induced in the adult liver-derived progenitor cell population (ALDPC) using Foxa2, HNF4α, and C/EBPα 96.

Unlike the classical signal flow of mutual crosstalk, simple, direct, and tiny molecular loops may be the mode in which HNF4α plays a partial role. Snail is a transcription factor known to repress the EMT epithelial program and often functions as a stem master regulator. In stable liver stem cell lines, Snail inhibits the program of differentiation toward the hepatocyte lineage by directly repressing HNF4α gene expression and the epithelial microRNAs (miR)-200c and -34a (which target binding to several stem cell genes to exert stemness repression, called stemness repressor microRNAs). The HNF4α KO mouse model shows that HNF4α can act as a transcriptional activator of microRNAs to inhibit the maintenance of cell stemness and promote the hepatic differentiation program. Molecular microcircuits of Snail, HNF4α, and microRNA interactions may control the stemness maintenance and differentiation trends of HPCs. In contrast, in hepatocytes, the first two are directly repressed to upregulate the transcription of microRNAs and ultimately stabilize the differentiation outcome of the hepatocyte phenotype 97, 98. Thus, a large spectrum of microRNAs such as miR-200c and -34a oscillate in response to the needs of the cellular program and its microenvironment, and specific cellular lineage control factors such as HNF4α are particularly important to ensure stable target differentiation 99.

In end-stage chronic liver disease, the transcription factor network in the liver is disrupted, including nuclear factor κB and HNF4α 100. To correct the defect in the transcription factor network, HNF4α forms a positive loop with its downstream transcription factors such as HNF1α, PPARα, C/EBPα, and FoxA2, which promote each other's upregulation. Therefore, HNF4α is considered to be the core and corrector of the transcription factor network in the adult liver. However, the study by Nishikawa *et al.* presented the opposite finding to the previous work in a model of end-stage liver disease 101. After intrahepatic HNF4α reprogramming was achieved by intravenous injection of viral particles, hepatocyte apoptosis was significantly suppressed, while the expression of HPCs markers CD44 and EpCAM was reduced. Based on such experimental results, the authors hypothesized that recovery from end-stage liver disease was not dependent on HPCs amplification and differentiation, and overexpression of HNF4α did not show a propensity effect on HPCs differentiation toward the hepatocyte lineage. Such an inference is questionable. It is not rigorous enough to determine the status of HPCs solely based on alterations in CD44 and EpCAM at the transcriptional level. We believe that the possible causes are the disruption of the transcription factor network by end-stage liver disease which leads to an incomplete correction of the numerous effector molecules regulated by HNF4α, or the occurrence of some negative feedback pathway because HPCs amplification exacerbates liver fibrosis, the exact mechanism of which remains to be investigated.

Inhibition of hepatocarcinogenesis by enforced expression of HNF4α has been extensively studied, and it attenuates hepatocyte EMT and alleviates liver fibrosis 102. In the cellular dimension, the mechanism of this effect is partly through HNF4α limiting the proliferation/migration capacity of HPCs to reduce the probability of malignant transformation of HPCs. Wang *et al.* found that migration was inhibited in the HNF4α overexpression model of HPCs (from on CDE diet rats). Cells were deposited around the portal vein after transplantation. Using ALB-positive hepatocyte filling as a criterion, the overexpression model was superior compared to the control group, and c-Myc (a transforming marker of invasive cancer cells) expression was reduced, but transplantation efficiency was poor 103. In addition, HNF4α with classical Wnt signaling may control liver zonated gene expression such as periportal (PP) and perivenular (PV). Resident liver stem cells (RLSCs) spontaneously acquire epithelial morphology and differentiate into PP hepatocytes. Transcriptionally driven by HNF4α, Wnt signaling convergence prompts PV gene activation and PP gene repression, resulting in the transformation of PP hepatocytes into PV hepatocytes 104.

Furthermore, the role of HNF4α may be also relevant in cholangiocarcinogenesis. Isocitrate dehydrogenase 1 (IDH1) mutations act on the HNF4α promoter P1 via targeted regulators to block transcription and block the differentiation of HPCs to the hepatocyte lineage without inhibiting biliary tract differentiation. Notably, after diethylnitrosamine (DEN, mimicking a second strike under IDH1 mutation) treatment, HPCs ablated by HNF4α continued to expand and showed similar morphology of intrahepatic cholangiocarcinoma (IHCC) 105. According to the aforementioned findings, specific changes in the initial commitment of HPCs brought on by disruption of transcription factors may be a source of carcinogenesis and HNF4α is a suppressor of a variety of intrahepatic cancers.

## HNF1: HPCs activation and tumor predisposition

Hepatocyte nuclear factor 1 (HNF1) is a highly differentiated homologous nuclear protein with a POU homologous structural domain and binds as a dimer to the promoter or enhancer of a target gene to activate transcription 106, 107. The HNF1 family includes two family members, HNF1α and HNF1β, located on chromosomes 12 and 17, respectively, and controls the transcription of a variety of organs, including the liver, intestine, pancreas, and kidney 108. HNF1 is also involved in the development and progression of several disorders of lipid metabolism and regulates hepatocyte homeostasis to attenuate the progression of nonalcoholic fatty liver disease (NAFLD) 106.

As mentioned above, Liver-enriched transcriptional factors are interwoven into a web, not just a unidirectional chain among transcription factors, and they often act reciprocally. Although HNF4α has a central position and initiating role in the transcriptional factor network 89, 92, 101, and the expression of HNF1 often occurs based on HNF4α activity, we still found that the regulatory effect of HNF1 is not weak. Binding sites for HNF1α/β, Sp1, HNF6, and GATA6 are present in the proximal promoter region of the HNF4α gene, and maintenance of high activity of the HNF4α promoter is dependent on synergistic interactions between them 109. Targeted perturbation of the HNF1β gene using siRNA revealed varying degrees of downregulation in all HNF families tested, including HNF1α, HNF3, HNF4α, and HNF6^110^. Such a conclusion is thought to be carried out with HNF1 as the top of the cascade hierarchy, which implies that the hierarchy of the Liver-enriched transcriptional factors network is constantly changing according to the molecular events of the cell, and even important as HNF4α cannot be fixed, their global position can be determined according to the degree of correlation and frequency of correlation with other transcription factors.

We suggest that HNF1 may be involved in ductal responses as one of the first transcription factors to promote HPCs activation. In chemical carcinogen-induced liver injury, mitosis of ductal cells and surrounding cells occurs rapidly, while expression of HNF1β and HNF3γ in ductal structures is immediately established 111. In our previously discussed study of oval cells, HNF1α, HNF1β, and HNF3γ were present in the bile ducts at the early stage of oval cell proliferation and were highly expressed together with HNF4 at a later stage 94. Based on the close association with the ductal response and the initial advantage, the upregulation of HNF1/3 may be an essential step for HPCs activation. Remarkably, HNF1β is one of the markers of mature bile duct cells, and Notch signaling activation upregulates HNF1β while downregulating HNF1α, implying that it is associated with the maintenance of hepatobiliary cell phenotype, precursor differentiation of biliary lineage, and self-renewal of adult stem cells 112, 113. Follow-up studies have shown that HPCs may be derived from HNF1β+ cell populations in the ductal response and that, following liver injury, HNF1β+ cell populations can cause expansion of HPCs to replenish periportal hepatocytes and contribute to liver regeneration, but this response is limited to specific liver injuries (from CDE diet mice) 114.

Similar to HNF4α, HNF1β is an important transcription factor for the maintenance of hepatic homeostasis, and its dysregulation is closely associated with epithelial-mesenchymal transition, metabolic reprogramming, and tumorigenesis 115, 116. HNF1β can regulate AFP promoter activity, which may play a specific role in recurrent hepatocellular carcinoma 117. High-throughput analysis of the cancer cell genome has identified HNF1α as a tumor suppressor and an important therapeutic target in hepatocellular carcinoma as well as HNF4α 115. Serrano *et al.* obtained bipotential iHepL cells using expression of reprogramming factors (Oct4, Sox2, Klf4, Myc) and liver fate conversion factors (HNF4α, HNF1α, and FoxA2), which, upon transplantation into the body, could differentiate into hepatocytes and bile duct cells, but some iHepL cells formed malignant non-teratoma cell aggregates. These tumor cells silenced key liver fate control factors, which included HNF1α 71.

## Conclusion

**Figure 3:**
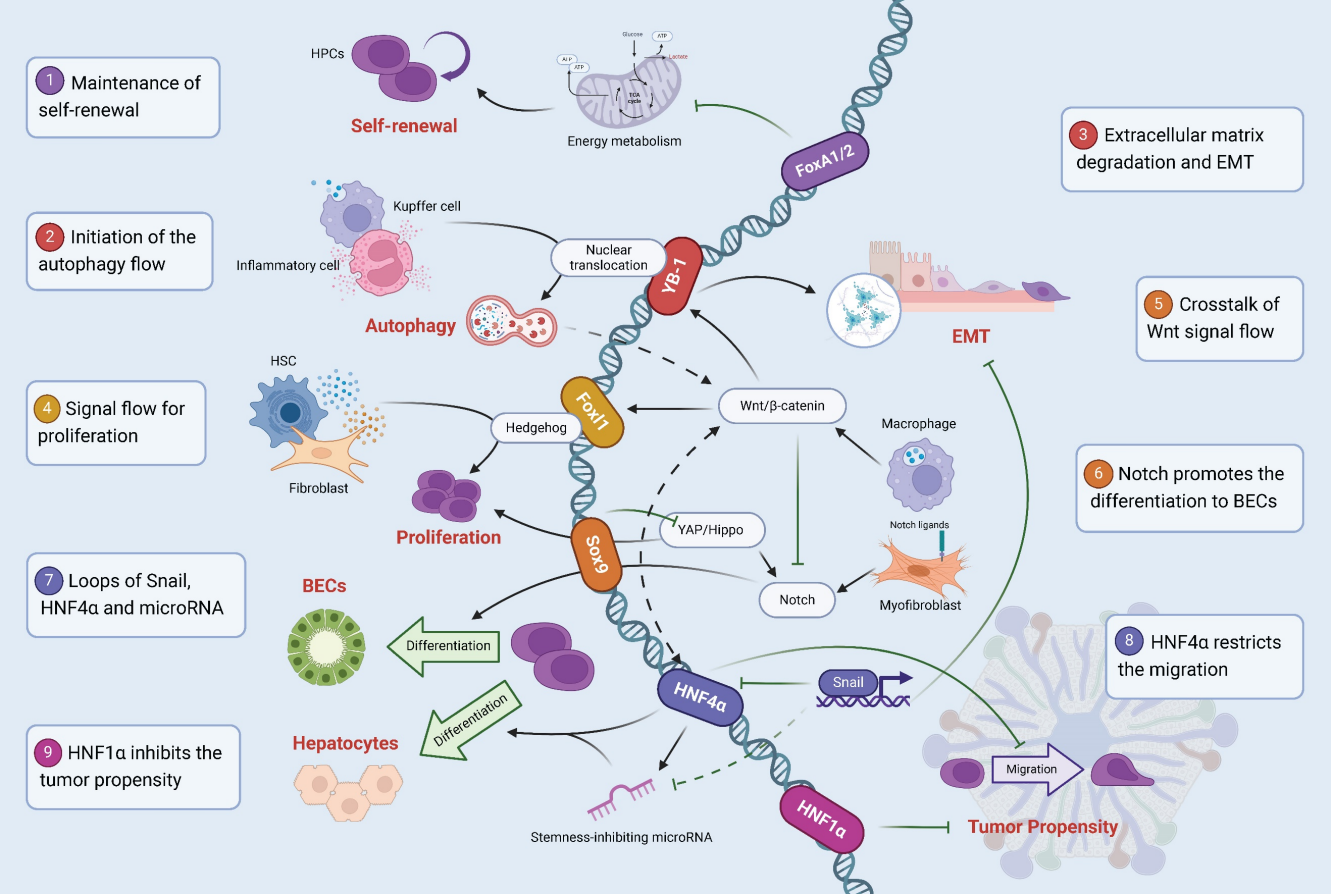
** Signal mapping of transcription factors regulating the fate of HPCs.** (1) FoxA1/2 inhibits cellular energy metabolism to maintain the self-renewal. (2) Kupffer cells and Inflammatory cells release TGF-β, induce YB-1 nuclear translocation, and initiate the autophagy flow. (3) YB-1 binds to Matrix metalloproteinase genes, degrades the extracellular matrix, and promotes EMT. (4) Foxl1 is involved in the maintenance of the proliferation by crosstalk fibroblasts and hepatic stellate cells through the Hedgehog signaling pathway. (5) Macrophages acquire Wnt signals after phagocytosis of hepatocyte fragments, damaging Notch to create hepatocyte specifications. (6) Notch ligands expression in myoblasts promotes the selection of HPCs to BECs. (7) Snail inhibits hepatocyte lineage differentiation by directly inhibiting HNF4α and microRNA. (8) HNF4α restricts the migration and reduces the probability of malignant transformation. (9) HNF1α inhibits the tumor propensity.

The fate regulation of adult HPCs is achieved by the synergistic directional effects of multiple signaling pathways and transcription factors **(Figure 3)**, which means that transcription factors are only one of many regulatory factors, and cytokines such as TNFα 118, Osteopontin (OPN) 119, epigenetic modifications, and non-coding regulatory elements, are also involved and play an important role. In addition, we found that it is difficult to unify the markers of adult HPCs in several studies, and specific labeling and identification is still a difficult task. The effective formation of a marker system and the identification of subpopulations of HPCs based on this is very valuable for the exploration of their internal heterogeneity and even the discovery of new intrahepatic cell populations.

The six classes of transcription factors (FoxA1/2/3, YB-1, Foxl1, Sox9, HNF4α, HNF1α, and HNF1β) discussed in this paper are well-recognized in the activation, selective differentiation, and tumorigenic changes of adult HPCs, but they are by no means limited to them. Their significance lies more in providing a research paradigm for the functional exploration and targeted intervention of more regulatory factors. In addition, transcription factor dysregulation is significant for the development of tumor predisposition in HPCs. Although we already know that the HNF family is a well-established tumor suppressor in the liver, the specific molecular mechanisms have not been elucidated. The world burden of hepatocellular carcinoma is severe 120, and how to utilize this potential molecular target to enhance their therapeutic function in intrahepatic tumors may be a pressing issue for the future.

## Figures and Tables

**Table 1:** Molecular characteristics of adult hepatic stem/progenitor cells in mouse model

Markers	Disease Model	References
CD133+/CD45-	DDC/ANIT/CCl_4_	10 11
CD24+/CD45-/Ter119-	DDC/Normal	12
EpCAM+/TROP2+	DDC	13
Foxl1+	DDC	14
Lgr5+	CCl_4_	15
Sox9+/HNF4α+	DDC	16, 17
CK19+	CDE/DDC	18
CK19+/ EpCAM+/A6+/OPN+/HNF4α+	Deficiency in FAH	19

ANIT, α-naphthylisothiocyanate; CCl_4_, carbon tetrachloride; CDE, choline-deficient, ethionine-supplemented diet; DDC, 3,5-diethoxycarbonyl-1,4-dihidro-collidine; FAH, fumarylacetoacetase.

## Abbreviations

ALDPC: adult liver-derived progenitor cell
ANIT: α-naphthylisothiocyanate
Atg7: autophagy-related gene 7
BDL: bile duct ligation
BECs: biliary epithelial cells
CCl_4_: carbon tetrachloride
CDE: choline-deficient, ethionine-supplemented diet
CoH: canals of Hering
CSD: cold shock domain
CTD: C-terminal domain
DDC: 3,5-diethoxycarbonyl-1,4-dihidro-collidine
DEN: diethylnitrosamine
DR: ductular reaction
DT: diphtheria toxin
ECM: extracellular matrix
EMT: epithelial-mesenchymal transition
FAH: fumarylacetoacetase
FoxA: forkhead box protein A
GSEA: Gene set enrichment analysis
HK2: hexokinase 2
HNF1: Hepatocyte nuclear factor 1
HNF4α: Hepatocyte nuclear factor 4 α
HPCs: Hepatic progenitor cells
IDH1: Isocitrate dehydrogenase 1
IHCC: intrahepatic cholangiocarcinoma
iHepL cells: Hepatic cells with progenitor-like features
LBD: ligand binding domain
LPLCs: liver-progenitor-like cells
MMPs: Matrix metalloproteinases
NAFLD: nonalcoholic fatty liver disease
OPN: Osteopontin
PHx: Partial hepatectomy
PI3K: phosphatidylinositol 3-kinase
PP: periportal
PV: perivenular
RLSCs: resident liver stem cells
Sox9: Sex-determining region Y-related high mobility group box9
TAZ: transcriptional co-activators with PDZ-binding motifs
YAP: Yes-associated protein 1
YB-1: Y-box binding protein-1
YFP: yellow fluorescent protein
